# Clinical implementation of deep learning-based automated left breast simultaneous integrated boost radiotherapy treatment planning

**DOI:** 10.1016/j.phro.2023.100492

**Published:** 2023-09-20

**Authors:** Michele Zeverino, Consiglia Piccolo, Diana Wuethrich, Wendy Jeanneret-Sozzi, Maud Marguet, Jean Bourhis, Francois Bochud, Raphael Moeckli

**Affiliations:** a Institute of Radiation Physics, Lausanne University Hospital and Lausanne University, Lausanne, Switzerland; b Radiation Oncology Department, Lausanne University Hospital and Lausanne University, Lausanne, Switzerland

**Keywords:** Treatment planning, Deep learning, Automation in radiation therapy, Breast cancer

## Abstract

**Background and purpose:**

Automation in radiotherapy treatment planning aims to improve both the quality and the efficiency of the process. The aim of this study was to report on a clinical implementation of a Deep Learning (DL) auto-planning model for left-sided breast cancer.

**Materials and methods:**

The DL model was developed for left-sided breast simultaneous integrated boost treatments under deep-inspiration breath-hold. Eighty manual dose distributions were revised and used for training. Ten patients were used for model validation. The model was then used to design 17 clinical auto-plans. Manual and auto-plans were scored on a list of clinical goals for both targets and organs-at-risk (OARs). For validation, predicted and mimicked dose (PD and MD, respectively) percent error (PE) was calculated with respect to manual dose. Clinical and validation cohorts were compared in terms of MD only.

**Results:**

Median values of both PD and MD validation plans fulfilled the evaluation criteria. PE was < 1% for targets for both PD and MD. PD was well aligned to manual dose while MD left lung mean dose was significantly less (median:5.1 Gy vs 6.1 Gy). The left-anterior-descending artery maximum dose was found out of requirements (median values:+5.9 Gy and + 2.9 Gy, for PD and MD respectively) in three validation cases, while it was reduced for clinical cases (median:−1.9 Gy). No other clinically significant differences were observed between clinical and validation cohorts.

**Conclusion:**

Small OAR differences observed during the model validation were not found clinically relevant. The clinical implementation outcomes confirmed the robustness of the model.

## 1 Introduction

Over the years, technological development in radiation therapy has brought innovation to both hardware and software tools with new irradiation techniques such as intensity-modulated radiation therapy (IMRT) and volumetric-modulated arc therapy (VMAT), and planning approaches (inverse planning, multi-criteria optimisation). While on the one hand this has led to an overall improvement of plan quality, on the other, the higher complexity of the planning process has engendered drawbacks like increased planning time or larger inter-planner variability [1], [2], [3], [4], [5].

The introduction of automation into radiation therapy has, among other advantages, a great potential to accelerate standardisation in treatment planning, thus minimising the aforementioned drawbacks affecting plan quality [6], [7]. Nowadays, different approaches are available for providing automated plans: from the simple use of dose objective templates [8], [9] to the more sophisticated techniques based on Artificial Intelligence (AI), and particularly on Machine Learning (ML). Among the ML-based techniques, commercial Knowledge-Based (KB) models utilise ML methods for learning the mapping of hand-crafted features extracted from the patient data to planning endpoints and constraints, such as the dose volume histogram (DVH) [10], [11], [12]. Such features may result in the loss of information, which potentially leads to a reduced predictive performance of the KB model limited for the regions of interest that are delineated [13], [14]. More recently, the introduction of Deep Learning (DL) techniques has circumvented this specific limitation of KB models since they do not rely on predefined rules (e.g. dose prediction for delineated organs at risk (OARs) only) but rather on their ability to automatically learn thousands of features from raw data and therefore, if properly trained, to predict a 3D-dose distribution for any given patient geometry and treatment site [15], [16], [17], [18], [19].

DL techniques were successfully employed in left-sided breast auto-planning for both early and locally advanced cancers [15], [19]. In both cases, dose prediction was restricted to a single dose prescription level (40.05 Gy) and exclusively for the IMRT treatment technique narrowing their range of applicability in breast cancer treatments.

In fact, several studies showed that around 70% of local recurrences (LR) occur mostly near or at the original tumour site [20], [21], [22], supporting the indication of the simultaneous integrated boost (SIB) to the tumour bed to reduce the risk of LR. Early breast cancer SIB treatments are more challenging for tangential IMRT techniques that may provide less dose conformity and homogeneity to both whole breast and boost planning target volumes (PTVs) than VMAT-based techniques [23]. The VMAT typical increase of low-dose spillage to the heart, may be mitigated treating the patients under deep inspiration breath-hold (DIBH) conditions providing the optimal planning dose trade-off between PTV and OARs [24].

To the best of our knowledge, no existing auto-planning solutions were available for early breast cancer SIB treatments. Furthermore, the previously mentioned models were not applicable due to their limitations in dose prescription and irradiation technique. Therefore, our goal was to train and develop a DL-based auto-planning model utilizing a VMAT technique. This model was specifically designed for left-sided early breast cancer patients receiving treatment under DIBH conditions.

## 2 Materials and methods

The DL auto-planning model was developed in collaboration with RaySearch Laboratories (RSL) for RayStation (RS) TPS v12A (RSL, Stockholm, Sweden) following four sequential steps: 1) data curation for model training, 2) model training, 3) model tuning, and 4) model validation. Step number 2 was entirely carried out by RSL, while in step number 3 RSL fine-tuned the mimicking parameters according to our clinical indications.

### 2.1 Data curation for DL model training

Initially, 60 left-sided breast patients treated at our institution under DIBH were included. Simulation CT and treatment planning were available for each patient. DIBH-CT resolution was 1x1x2 mm^3^. Two 6FFF MV reversed partial arcs (span range 210°-230°) from an Elekta Agility linear accelerator (Elekta AB, Stockholm, Sweden) were employed. Start (range 285°- 310°) and stop (range 135° − 160°) gantry angles were manually chosen to minimise contra-lateral breast irradiation according to the patient’s anatomy. Collimator angles were set to 5° and 355°, respectively. For each arc, control points were defined every 3° resulting in a segment range varying from 70 to 77 and maximum allowed beam-on time was 75 s. Dose calculation grid (Collapsed Cone Convolution (CCC) algorithm) was 3x3x3mm^3^. The SIB and whole-breast planning target volumes (PTV_Boost and PTV_Breast, respectively) were generated by expanding their respective clinical target volumes by 5 mm and then cropping it 3 mm under the skin. Prescription doses of 60 Gy and 50 Gy were simultaneously delivered in 25 fractions for the PTV_Boost and PTV_Breast, respectively, during four to six breath-hold cycles.

Original clinical plans (C-Plans) were randomly optimized by five medical physicists using different dose-volume optimization objectives templates and following the RTOG 1005 protocol [25] for dose-volume constraints. Patient-specific QA, performed with the Octavius II phantom and analysed with the Verisoft software (PTW, Freiburg, Germany), returned gamma-pass value > 95% of points with 3%/3mm criteria (no dose scaling and dose difference normalised to global dose maximum) for each plan.

To reduce the inter-operator variability and improve the quality of the existing dose distributions, data curation was approached as follows. For each patient, OAR contours were reviewed and adjusted when needed, and the dose was recomputed. A list of clinical goals was extracted from each plan and the median value of each clinical goal over the 60 plans was used to define a new list of clinical goals to be achieved (Table 1).

**Table 1:** List of clinical goals to be achieved for plans used as model training data. PTV2_Crop was the difference without margins between PTV_Breast and PTV_Boost. LAD is the abbreviation for Left Anterior Descending Artery.

Structure	Dose-Volume Objective	Requirement
PTV_Boost	D98%	≥ 57 Gy
	D2%	≤ 61.8 Gy
	V57Gy	≥ 96%
	V56Gy	≥ 99%
	V61.8 Gy	≤ 2%
PTV_Breast	V47.5 Gy	≥ 95%
	V46.5 Gy	≥ 98%
PTV_Crop	D1%	≤ 60 Gy
	V52.5 Gy	≤ 15%
Lung_L	V5Gy	≤ 33%
	V10Gy	≤ 20%
	V20Gy	≤ 10%
	V40Gy	≤ 2%
	Dmean	≤ 7 Gy
Lung_R	D1%	≤ 5 Gy
	Dmean	≤ 1.5 Gy
Heart	D1%	≤ 6 Gy
	Dmean	≤ 1.5 Gy
Breast_R	D1%	≤ 9 Gy
	Dmean	≤ 2 Gy
LAD	Dmax	≤ 8 Gy

Fifty-two out of the 60 C-plans were reoptimized due to different reasons: 1) 45/52 because of missing structures or objectives of secondary importance, 2) 28/52 because of sub-optimal choice of optimization objectives for OARs listed in Table 1, and 3) 15/52 because not complying with the dosimetry protocol of Table 1. In the first case, missing objectives controlling both mean and maximum doses were introduced. In the second one, the maximum and mean dose objectives were added or revised in their formulation for specific OARs listed in Table 1. In the last case the whole list of optimization objectives was revised. The planning comparison between plans used for model training and original clinical plans is reported in Fig. S1(a),(b), and Table S1 of Supplementary Material.

To improve the heterogeneity of the input data for model training, 20 new additional plans were optimised according to the clinical goals listed in Table 1 for a total of 80 plans used for model training (MT-plans). Fig. S2 of the Supplementary Material presents the distribution of the PTV_Breast volumes of the patients used for model training.

The same dosimetric protocol of Table 1 and planning parameters were finally used to generate 15 new additional manual plans to be used for model tuning (5) and validation (10).

According to local regulations, there was no need for ethical and/or legal approval for the present study.

### 2.2 DL model training, predicted and mimicked dose

The DL technique used for model training was based on the U-Net convolutional neural network (CNN) [26]. Briefly, through sequential convolutional and de-convolutional layers it was able to incorporate both local and global features for learning a pixel-to-pixel mapping between imaging and dose data to predict the 3D dose distribution for any given 3D anatomical data [27], [28]. Predicted dose distribution was not directly exploitable as it needed to undergo the mimicking process to become clinically applicable through an optimization process. In RS, DL predicted dose serves as reference input for the voxel-based objectives of the dose mimicking process. Goals and constraints applied as post-processing to the predicted dose give rise to different reference inputs and, similarly, varying the mimicking objectives different outcomes are possible. The set of instructions used for dose mimicking was iteratively adjusted by RSL during the model tuning under our supervision to fulfil the clinical goals of Table 1 and released as an instruction file in the JavaScript Object Notation (JSON) format readable by RS. Although mimicked dose can be tailored to a specific list of clinical goals by altering the objectives, the same set of dose mimicking objectives and weights as released were used for all patients. This involved three intermediate CCC dose calculations: two over the course and one at the end of the 180 dose mimicking iterations.

### 2.3 DL model tuning

Auto-planning model was initially evaluated comparing auto- with manual plans for five new patients. Given the predicted dose, the tuning phase involved modifications to the JSON file only. Two senior medical physicists and one expert radio-oncologist performed a blind evaluation according to their clinical experience. A five-value scoring scale was used to compare the plans generated by the DL-based model (DL-plans) and the test plans (T-plans): 1.Worse, 2.Slightly worse, 3.Equivalent, 4. Slightly better, 5. Better. The model was considered acceptable for validation only if the scores were ≥ 3 for both PTVs and all OARs involved.

### 2.4 DL model validation

For each clinical goal in Table 1, predicted and mimicked outcomes were evaluated against the corresponding manual result by the percent error (PE) calculated as $\frac{\mathrm{DLclinicalgoal}-Manualclinicalgoal}{\mathrm{Manualclinicalgoal}}∙100$. Paired Wilcoxon signed-rank tests were performed to assess statistically significant differences (p < 0.05) for predicted and mimicked dose PE.

In addition, a blind comparison between manual and auto-plans was carried out by an experienced radiation oncologist by means of the same scoring scale reported in the previous paragraph.

### 2.5 DL model clinical evaluation

The model was employed to generate 17 clinical plans after its validation. Clinical and validation cohorts were compared in terms of dose distribution to assess statistically significant differences using the Wilcoxon rank-sum test because of different size between samples. Furthermore, the achievement of the clinical goals requirements was also investigated.

## 3 Results

### 3.1 DL model tuning

**Fig. 1:**
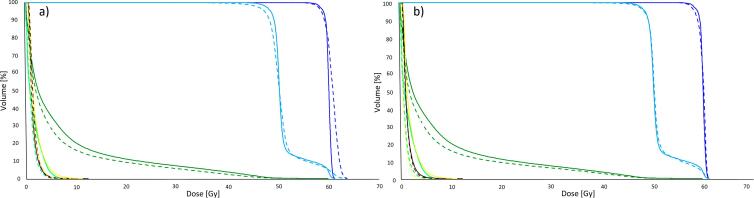
DVH comparison between manual (solid lines) and auto-(dashed lines) plans for the initial (a) and the final (b) version of the model for a test case. It is clearly visible for the initial model the over-dosage of the PTV1_Boost (dark blue) and the under-dosage of PTV_Breast (light blue). The final model equalised the manual dose to the PTVs while keeping the same less dose to the left (dark green) and right (light green) lung. Right breast (yellow) and heart (brown) were unchanged between the two versions with respect to the manual dose. (For interpretation of the references to colour in this figure legend, the reader is referred to the web version of this article.)

For the initial version of the DL model only one out of the five test patients had an equivalent score for PTVs while all DL-plans were better or at least equivalent for OARs. This was due to the over-sparing of the OARs, particularly for the left lung, causing the loss in homogeneity for both PTVs. Therefore, the model was progressively improved by tuning the mimicking parameters defined in the set of model instructions. Dose constraints to the left lung and contralateral breast were systematically relaxed, enabling a better PTV homogeneity as shown in Fig. 1.

The fourth version of the model was accepted for validation as all DL-plans were equivalent or better than T-plans (see Table S2).

### 3.2 DL model validation

Predicted dose median values met all clinical goals. For PTVs, mean doses were well aligned to manual plans (PE < 1%), while PTV_Breast coverage resulted significantly improved (PE = 1.1%). For OARs, large PE values were not correlated with clinically significant differences of median values. As expected, the mimicking process altered the initial dose prediction. Mimicked dose resulted worse than predicted dose for PTV_Boost maximum dose and PTV_Breast coverage. On the other hand, it was found significantly better in terms of OAR dose sparing, especially for left lung and contralateral breast.

Concerning the comparison between mimicked and manual plans, PTVs differences were not significant except for the PTV_Crop D1% showing an improving of dose conformity for auto-plans. For OARs, auto-plans returned better results for both lungs and contra-lateral breast while manual plans were superior in terms of heart dose sparing (see Table 2 and Fig. S3(a),(b) for details).

**Table 2:** Planning comparison between manual, predicted and mimicked dose averaged over the 10 validation patients.

Structure	Figure of Merit	Unit	Manual Dose		Predicted Dose		Mimicked Dose		PE (%)		p- value	
			Median	Range	Median	Range	Median	Range	Predicted	Mimicked	Predicted	Mimicked
PTV_Boost	D98%	Gy	57.2	56.5–58.1	57.1	55.5–57.7	57.1	56.8–57.4	−0.2	−0.2	0.38	0.11
	D2%	Gy	61.4	61.1–62.3	61.3	61.1–61.6	61.4	61.1–62.2	−0.1	0.0	0.28	0.92
	V57Gy	%	98.6	97.1–99.6	98.3	96.6–99.1	98.4	97.3–98.8	−0.3	−0.2	0.23	0.19
	V56Gy	%	99.6	98.7–99.9	99.4	97.7–99.8	99.5	99.2–99.8	−0.3	−0.1	0.32	0.56
	V61.8 Gy	%	0.6	0–5.5	0	0–0.7	0.1	0–7.3	−100.0	−81.4	0.02	0.91
	Dmean	Gy	59.9	59.7–60.0	59.7	59.5–59.9	59.8	59.6–59.9	−0.3	−0.2	0.01	0.24
PTV_Breast	V47.5 Gy	%	95.7	95.4–96.8	96.7	96.1–97.6	96.0	94.3–97.0	1.1	0.4	0.01	1
	V46.5 Gy	%	97.5	95.4–96.8	98.0	97.5–98.5	97.8	96.2–98.3	0.6	0.3	0.02	0.92
	Dmean	Gy	51.2	50.8–52.1	50.8	50.6–52.3	51.3	50.7–52.5	−0.8	0.2	0.04	0.18
PTV_Crop	D1%	Gy	58.6	58.0–59.5	58.2	57.4–59.8	57.8	57.2–58.5	−0.6	−1.3	0.23	< 0.01
	V52.5 Gy	%	10.8	8.4–19.3	8.0	5.9–19.6	10.5	7.1–17.6	−25.8	−2.3	0.11	0.32
Lung_L	V5Gy	%	30.9	19.1–39.7	29.4	21.1–36.4	23.1	18.1–28.6	−4.8	−25.3	0.32	< 0.01
	V10Gy	%	18.1	10.9–23.6	16.0	7.9–22.1	13.4	7.6–17.8	−11.5	−25.7	0.23	< 0.01
	V20Gy	%	8.9	3.0–11.3	8.3	2.4–12.5	7.3	2.5–10.5	−6.5	−17.6	0.85	< 0.01
	V40Gy	%	1.3	0–1.9	1.3	0–2.7	1.2	0–2.2	6.1	−4.4	0.11	0.19
	Dmean	Gy	6.1	4.1–7.4	5.9	3.7–7.6	5.1	3.5–6.4	−2.5	−15.9	0.56	< 0.01
Lung_R	D1%	Gy	3.6	1.6–6.4	3.6	2.3–5.5	3.4	1.7–5.0	−0.5	−6.8	0.63	0.11
	Dmean	Gy	0.9	0.5–1.6	1.0	0.7–1.4	0.9	0.5–1.2	1.6	−8.4	0.7	0.08
Heart	D1%	Gy	4.6	3.5–5.5	5.2	3.6–6.1	5.4	4.2–6.1	13.2	18.0	0.19	< 0.01
	Dmean	Gy	1.4	1.1–1.5	1.5	1.2–2.0	1.5	1.2–1.7	4.1	4.1	0.43	< 0.01
Breast_R	D1%	Gy	5.2	2.5–12.0	5.9	3.0–10.0	5.2	2.2–9.1	13.9	−0.2	0.85	< 0.01
	Dmean	Gy	1.1	0.8–2.8	1.5	0.9–2.4	1.2	0.5–2.0	46.1	12.4	0.23	0.38
LAD	Dmax	Gy	7.1	4.6–12.5	7.8	4.9–16.6	7.7	5.6–12.4	9.1	7.6	0.11	0.38

Individual plan analysis showed that predicted dose failed to achieve 35 clinical goals out of the 210. In all cases, deviations were negligible or not clinically significant except for the LAD maximum dose that resulted overpredicted for three cases ranging from 5.2 Gy to 8.6 Gy. After mimicking, the number of failed clinical goals were reduced to 20 and the LAD overdosage range halved (see Table 3). The achievement of clinical goals listed per plan is reported in Table S3 of supplementary material.

**Table 3:** Evaluation of model failed objectives and corresponding deviation with respect to the requirement listed for both predicted and mimicked dose for the 10 validation patients.

	Objective		Failed objectives					
Structure	Figure of Merit	Requirement	Predicted Dose			Mimicked dose		
			Number	Deviation		Number	Deviation	
				Median	Range		Median	Range
PTV1	D98%	≥ 57 Gy	2	−1.2 Gy	(-1.5 - −0.8) Gy	1	−0.2 Gy	–
	D2%	≤ 61.8 Gy	0	–	–	1	0.4 Gy	–
	V57Gy	≥ 96%	0	–	–	0	–	–
	V56Gy	≥ 99%	3	–	–	0	–	–
	V61.8 Gy	≤ 2%	0	–	–	1	5.3 %	–
PTV2	V47.5 Gy	≥ 95%	0	–	–	2	−0.4 %	(-0.7 - −0.1) %
	V46.5 Gy	≥ 97%	0	–	–	2	−0.5 %	(-0.8 - −0.2) %
PTV_Crop	D1%	≤ 60 Gy	0	–	–	0	–	–
	V52.5 Gy	≤ 15%	1	4.6 %	–	1	2.6 %	–
Lung_L	V5Gy	≤ 33%	3	2.6 %	(0.8–3.3) %	0	–	–
	V10Gy	≤ 20%	3	1.3 %	(1.1–1.6) %	0	–	–
	V20Gy	≤ 10%	3	1.7 %	(1.7–2.6) %	2	0.4 %	(0.3–0.5) %
	V40Gy	≤ 2%	3	0.8 %	(0.3–0.9) %	2	0.2 %	(0.1–0.2) %
	Dmean	≤ 7 Gy	3	0.4 Gy	(0.2–0.7) Gy	0	–	–
Lung_R	D1%	≤ 5 Gy	2	0.4 Gy	(0.1–0.6) Gy	0	–	–
	Dmean	≤ 1.5 Gy	0	–	–	0	–	–
Heart	D1%	≤ 6 Gy	1	0.1 Gy	–	1	0.1 Gy	–
	Dmean	≤ 1.5 Gy	4	0.3 Gy	(0.2–0.5) Gy	3	0.1 Gy	–
Breast_R	D1%	≤ 9 Gy	1	1 Gy	–	1	0.1 Gy	–
	Dmean	≤ 2 Gy	3	0.5 Gy	(0.3–0.5) Gy	0	–	–
LAD	Dmax	≤ 8 Gy	3	5.3 Gy	(5.2–8.6) Gy	3	2.9 Gy	(2.2–4.4) Gy

Blind comparison evaluation provided results in support of auto-planning: auto-plans were judged equivalent, slightly better and better than manual plans twice, six times and twice, respectively. The main reason of such preference was the less dose to the left lung.

### 3.3 Clinical evaluation

Across the cohorts, the clinical and validation auto-plans were found well aligned except for the PTV_Crop D1% and LAD maximum dose that resulted significantly higher (+0.5 Gy) and lower (-1.9 Gy), respectively, for clinical plans as reported in Table 4.

**Table 4:** Planning comparison between clinical and validation cohorts (note that results referred to different patients).

Structure	Figure of Merit	Unit	Clinical Plans (n = 17)		Validation Plans (n = 10)		p- value
			Median	Range	Median	Range	
PTV_Boost	D98%	Gy	57.3	56.6–57.7	57.1	56.8–57.4	0.17
	D2%	Gy	61.5	61.0–62.6	61.4	61.1–62.2	0.36
	V57Gy	%	98.7	97.1–99.3	98.4	97.3–98.8	0.16
	V56Gy	%	99.7	99.1–100	99.5	99.2–99.8	0.44
	V61.8 Gy	%	0.3	0–9.9	0.1	0–7.3	0.92
	Dmean	Gy	59.9	59.6–60.1	59.8	59.6–59.9	0.02
PTV_Breast	V47.5 Gy	%	96.0	95.3–97.3	96.0	94.3–97.0	0.86
	V46.5 Gy	%	97.7	97.1–98.4	97.8	96.2–98.3	0.82
	Dmean	Gy	51.3	50.4–51.7	51.3	50.7–52.5	0.92
PTV_Crop	D1%	Gy	58.3	57.6–58.8	57.8	57.2–58.5	< 0.01
	V52.5 Gy	%	11.5	5.5–15.2	10.5	7.1–17.6	0.57
Lung_L	V5Gy	%	24.7	18.5–30.9	23.1	18.1–28.6	0.68
	V10Gy	%	14.2	8.9–19.2	13.4	7.6–17.8	0.68
	V20Gy	%	8.2	4.0–12.1	7.3	2.5–10.5	0.71
	V40Gy	%	1.5	0.2–2.5	1.2	0–2.2	0.50
	Dmean	Gy	5.4	3.9–7.0	5.1	3.5–6.4	0.68
Lung_R	D1%	Gy	3.8	2.2–6.3	3.4	1.7–5.0	0.39
	Dmean	Gy	1.0	0.5–1.4	0.9	0.5–1.2	0.39
Heart	D1%	Gy	4.6	3.3–6.5	5.4	4.2–6.1	0.24
	Dmean	Gy	1.6	1.0–1.8	1.5	1.2–1.7	0.24
Breast_R	D1%	Gy	4.5	2.3–7.7	5.2	2.2–9.1	0.41
	Dmean	Gy	1.1	0.7–2.0	1.2	0.5–2.0	0.64
LAD	Dmax	Gy	5.8	3.4–8.0	7.7	5.6–12.4	< 0.01

Clinical goals were not fulfilled in 27 out of 357 total evaluations providing similar results to the validation plans in terms of failing percentage (8.4% vs 7.5%) and negligible clinical impact (see Table 5). Detailed clinical goal evaluation is reported in Table S4 of Supplementary Material.

**Table 5:** Evaluation of model failed objectives and corresponding deviation with respect to the requirement for the 17 clinical patients.

	Objective		Failed objectives		
			Clinical Cases (Mimicked Dose)		
Structure	Figure of	Requirement			
			Number	Deviation	
				Median	Range
PTV1	D98%	≥ 57 Gy	3	−0.1 Gy	(-0.4 - −0.1) Gy
	D2%	≤ 61.8 Gy	2	0.7 Gy	(0.6–0.8) Gy
	V57Gy	≥ 96 %	0	–	–
	V56Gy	≥ 99 %	0	–	–
	V61.8 Gy	≤ 2 %	2	5.8 %	(3.8–7.7) %
PTV2	V47.5 Gy	≥ 95 %	0	–	–
	V46.5 Gy	≥ 97%	0	–	–
PTV_Crop	D1%	≤ 60 Gy	0	–	–
	V52.5 Gy	≤ 15 %	1	0.2 %	–
Lung_L	V5Gy	≤ 33 %	0	–	–
	V10Gy	≤ 20 %	0	–	–
	V20Gy	≤ 10 %	1	0.1 %	–
	V40Gy	≤ 2 %	4	0.3 %	(0.1–0.5) %
	Dmean	≤ 7 Gy	0	–	–
Lung_R	D1%	≤ 5 Gy	3	0.2 Gy	(0.1–1.3) Gy
	Dmean	≤ 1.5 Gy	0	–	–
Heart	D1%	≤ 6 Gy	3	0.4 Gy	(0.1–0.5) Gy
	Dmean	≤ 1.5 Gy	8	0.1 Gy	(0.1–0.2) Gy
Breast_R	D1%	≤ 9 Gy	0	–	–
	Dmean	≤ 2 Gy	0	–	–
LAD	Dmax	≤ 8 Gy	0	–	–

## 4 Discussion

This study reported on the clinical implementation of a new DL-based auto-planning model for VMAT left-breast treatment under DIBH. Overall, 95 different patients were involved to conduct and validate the model that afterwards was successfully clinically applied for 17 patients.

The predict mean dose error for PTVs and OARs was well aligned to previous findings using similar CNN architecture and treatment site [15], [19] confirming the accuracy of the U-net in predicting dose for large structures. On the other hand, the LAD maximum dose showed large variations in dose prediction. This might be due to the increased uncertainty in predicting the dose within few voxels lying on the dose gradient region, therefore in this case the use of a more robust metrics such as near-maximum dose for dose reporting would be of help.

Dose mimicking significantly improved the OAR dose sparing. The auto-planning workflow involved a predicted dose post-processing before undergoing the dose mimicking process [19], [29]. The magnitude of the post-processing was defined during the model tuning phase by exploring the dosimetric trade-offs achievable from the initial predicted dose. It aimed to intentionally alter the predict dose towards a specific trade-off that could produce clinically realistic dose distribution once mimicked. Specifically, left lung and right breast sparing was privileged over a less (but still within the evaluation criteria) PTV_Boost homogeneity and PTV_Breast coverage.

Post-processing of predicted dose may be considered as a powerful tool able to improve training data of any quality. Although this is true to some extent, a revised set of training data maximizes the efficiency of the process. In fact, this allowed the optimal explorations of trade-offs from the original predicted dose where dosimetric outliers were due to patient geometrical variation only [30]. Furthermore, post-processing applied user-defined dose reduction functions that have an impact on the whole structure acting as a dose re-normalization weight. Hence, without data curation, the post-processing would have reduced the overall predicted dose keeping the same level of heterogeneity of training data. However, the large dose difference observed between predicted and mimicked dose for the left lung and right breast, suggested that during data curation the dose trade-off for that OARs was not fully explored.

After mimicking, the DL auto-planning model provided good results as all the clinical goals were met. When compared to our clinical plans it performed better in terms of PTV dose conformity and OAR sparing except for the heart. The model was built so that a small penalty was accepted for the heart (median dose: +0.1 Gy, D1% +0.9 Gy) to better spare the left lung (median dose: −1Gy). This trade-off was acceptable as both the maximum and mean heart dose were still within the clinical goal.

All automatic plans could have been further optimised after automatic optimisation by the addition of new dose-volume objectives just like for standard plans [15], [19]. This could have solved the slight overdosage of the heart. However, we evaluated the results for auto-plans without any further optimization as we were looking for a fully automated solution.

Once adopted in the clinical practice, the model provided only two significant differences with respect to the validation cases: the increase of PTV_Crop D1% and LAD dose sparing. The average PTV_Boost volume was found for the clinical cohort slightly larger than for validation cohort (59 cm^3^ vs 51 cm^3^), probably explaining the increase of dose received by the PTV_Crop. The higher average value of LAD maximum dose observed for the validation cohort was due to three dose outliers (see individual plan evaluation in the supplementary material) corresponding to patients with reduced DIBH capabilities.

Obviously, the model presented here was tailored to our clinical practice. It may not reflect the clinical standards of other centres where different treatment protocols are used. Nonetheless, the automated solution implemented in RS enables the user to drive the optimisation towards different solutions, given the predicted dose from the U-net CNN. As mentioned, by acting on the set of instructions of the predicted and the mimicked dose, as well as by editing the set of dose-volume objectives, it is possible to modify the resulting dose distribution. New strategies may involve the extra-sparing of whichever OAR needed, for instance in case of a previous irradiation of the right breast. However, any new model modification needs to pass an internal qualitative and quantitative evaluation.

Since plan quality and treatment-related toxicity are strongly dependent upon breast volume [31], [32], it is often taken as an indicator of anatomic differences [33], [34]. A potential limitation of the study was the range of whole breast volume of the model validation group of patients (605 cm^3^ – 876 cm^3^) with respect to the range of the training patients (193 cm^3^ – 1565 cm^3^). Therefore, model validation was *a fortiori* limited to a narrow range of clinical cases. Clinical cases extended the whole breast volume range (186 cm^3^ – 1260 cm^3^), showing the robustness of the model for small and medium-sized breast volumes.

The behaviour for large-sized breasts (>1200 cm^3^) remains to be evaluated, although a series of different DL approaches showed model robustness with respect to anatomical variation for other treatment sites [35]. Nonetheless, the use of beam energies higher than 6MV may still provide better outcomes in terms of dose homogeneity for large breast volumes.

In conclusion, a new DL-based automated planning solution for left-sided SIB breast treatments under DIBH was developed and successfully implemented in clinical routine filling the existing gap for this specific clinical indication.

## CRediT authorship contribution statement

**Michele Zeverino:** Conceptualization, Data curation, Formal analysis, Investigation, Methodology, Validation, Writing – original draft, Writing – review & editing. **Consiglia Piccolo:** Validation, Writing – review & editing. **Diana Wuethrich:** Software, Writing – review & editing. **Wendy Jeanneret-Sozzi:** Validation, Writing – review & editing. **Maud Marguet:** Validation, Writing – review & editing. **Jean Bourhis:** Methodology, Validation, Writing – review & editing. **Francois Bochud:** Methodology, Formal analysis, Supervision, Validation, Writing – review & editing. **Raphael Moeckli:** Conceptualization, Methodology, Formal analysis, Supervision, Validation, Writing – original draft, Writing – review & editing.

## Declaration of Competing Interest

The authors declare that they have no known competing financial interests or personal relationships that could have appeared to influence the work reported in this paper.
