# Supplementary material for: Clinical implementation of deep learning-based automated left breast simultaneous integrated boost radiotherapy treatment planning
Source: Phys Imaging Radiat Oncol. 2023 Sep 20;28:100492. doi: 10.1016/j.phro.2023.100492 (PMC10534254; doi:10.1016/j.phro.2023.100492)
Supplement: Supplementary data 1 [file mmc1.docx]

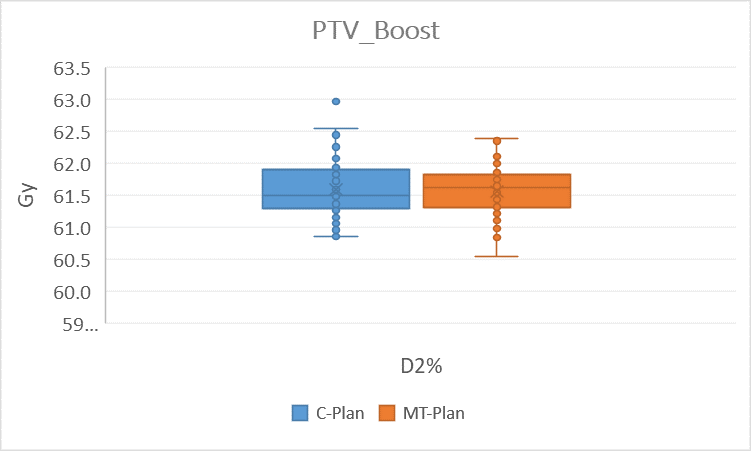

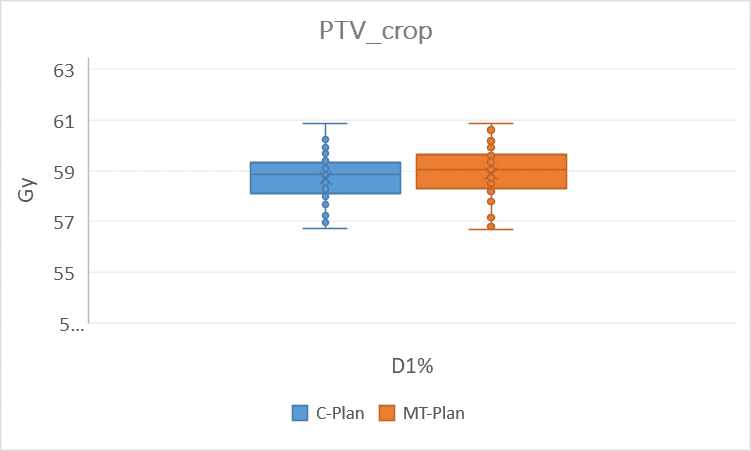

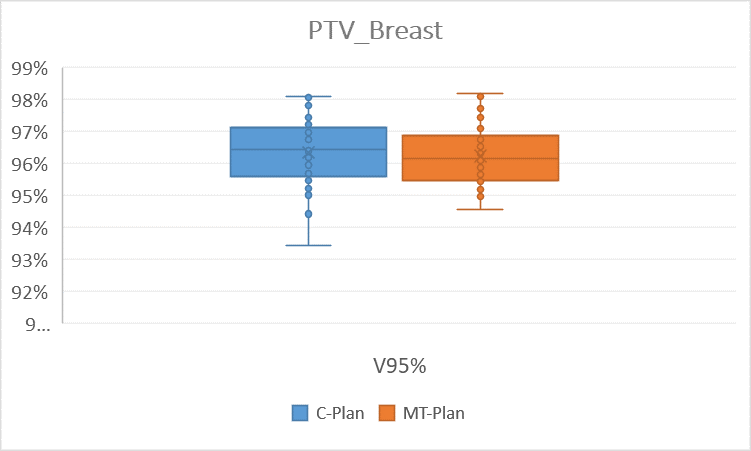

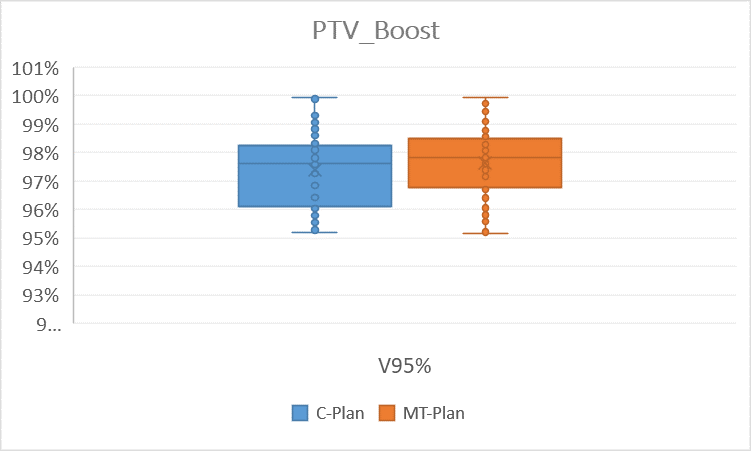

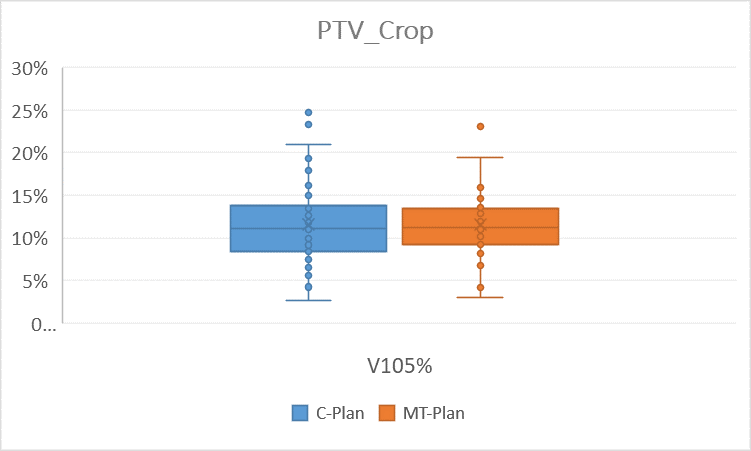

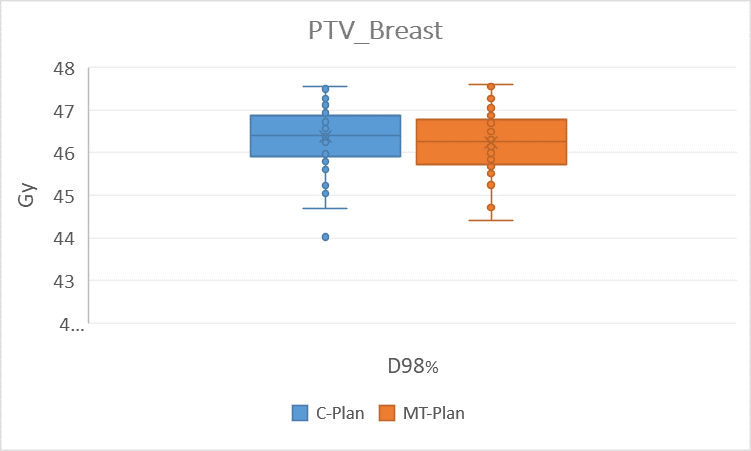

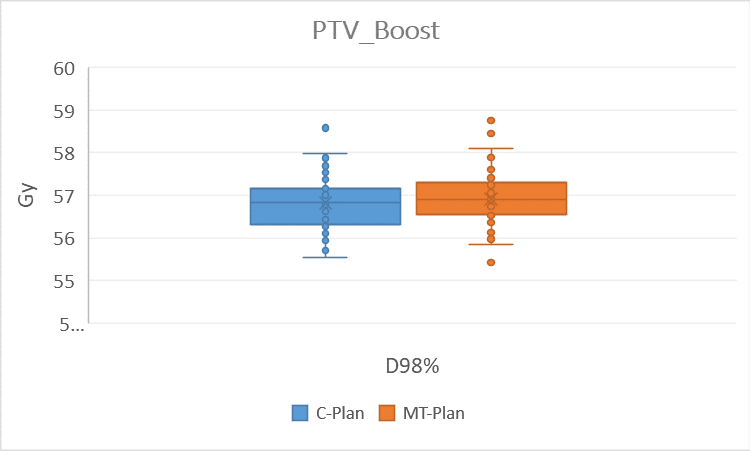
**Supplementary materials**

Figure 1s (a). Box plots dose comparison between the original clinical plans (C-Plans, depicted in blue) and plans used for model training (MT-Plans, depicted in orange) for targets. The dots represent the clinical goal value for each plan, while the straight horizontal line, and the cross in the box represent the median and the mean values of the dose distributions (60 cases for each plan cohort), respectively.


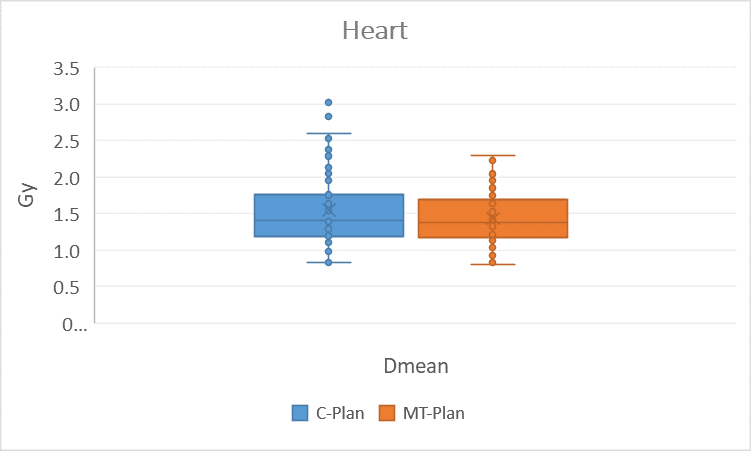

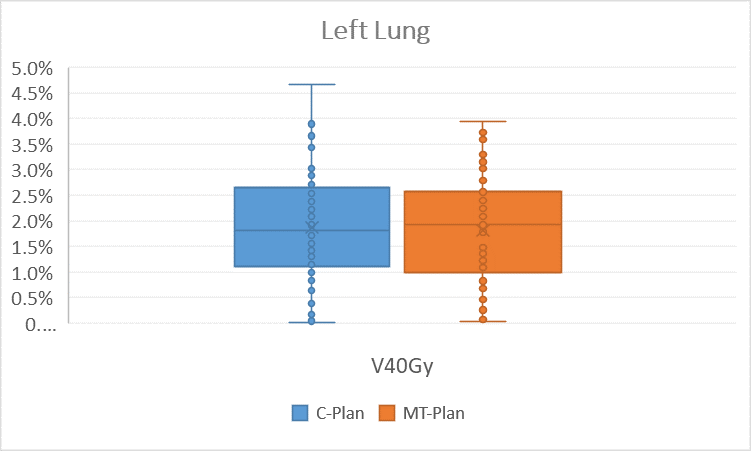

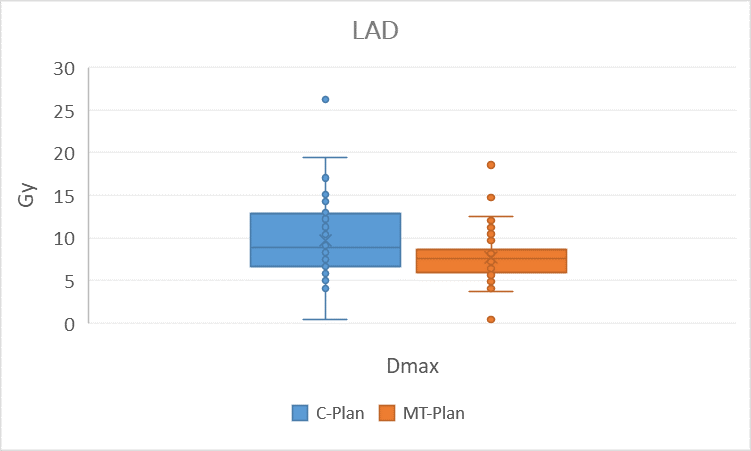

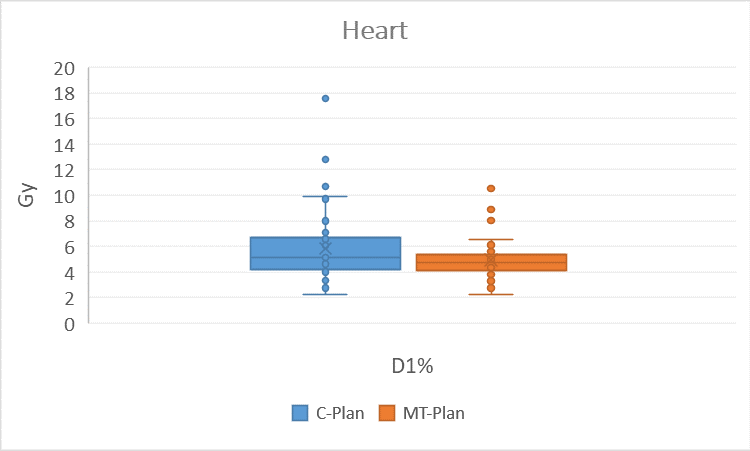

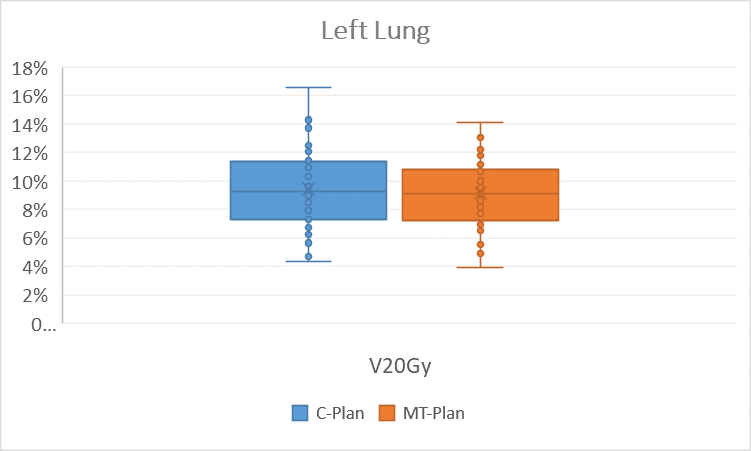

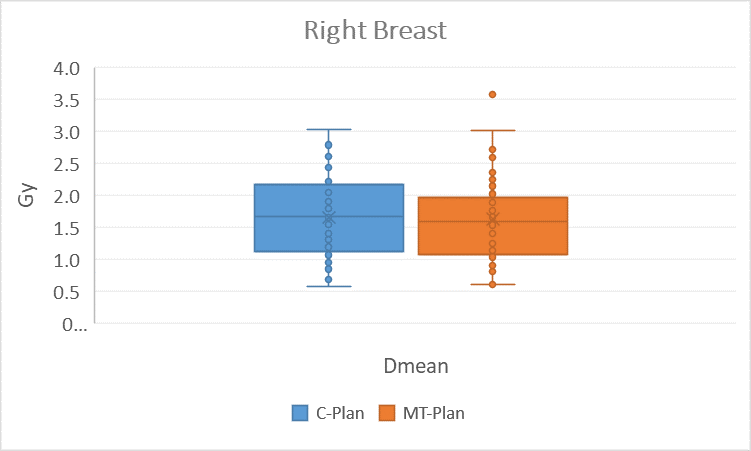

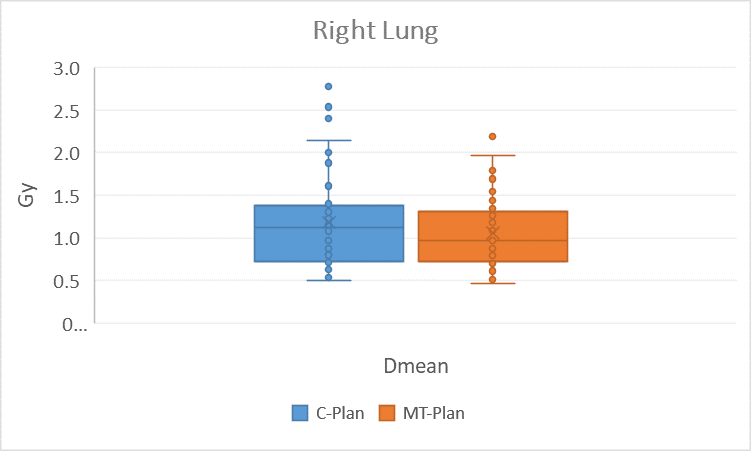

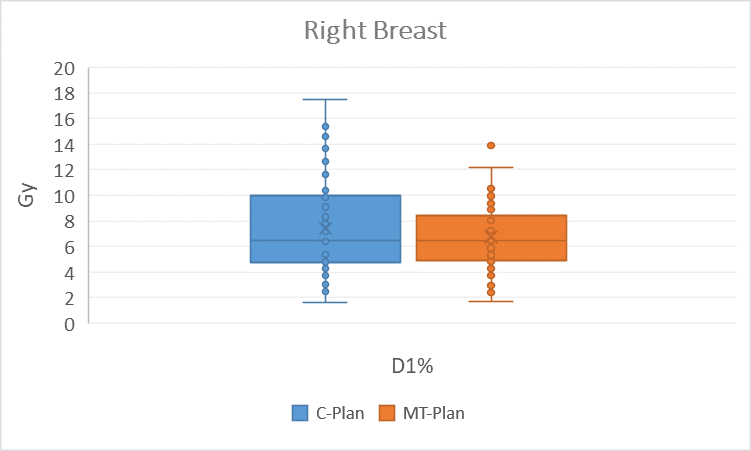

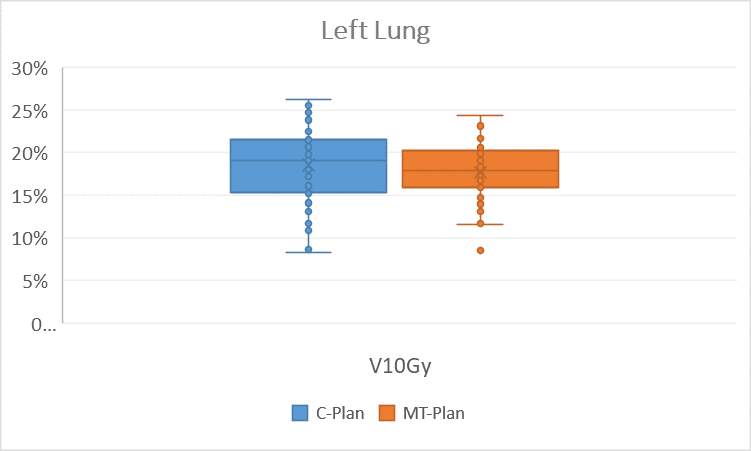

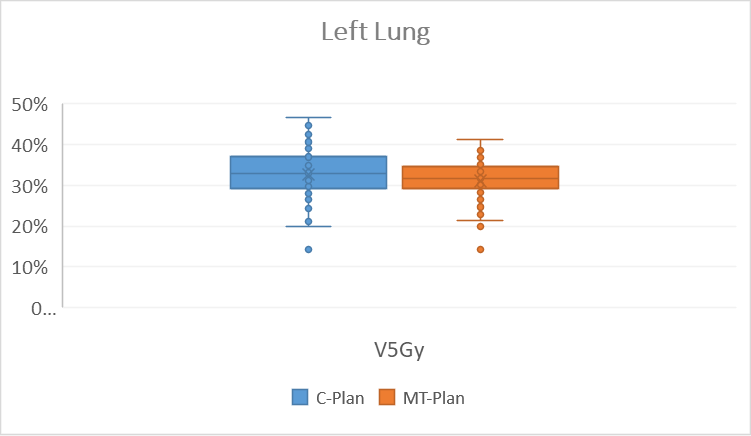

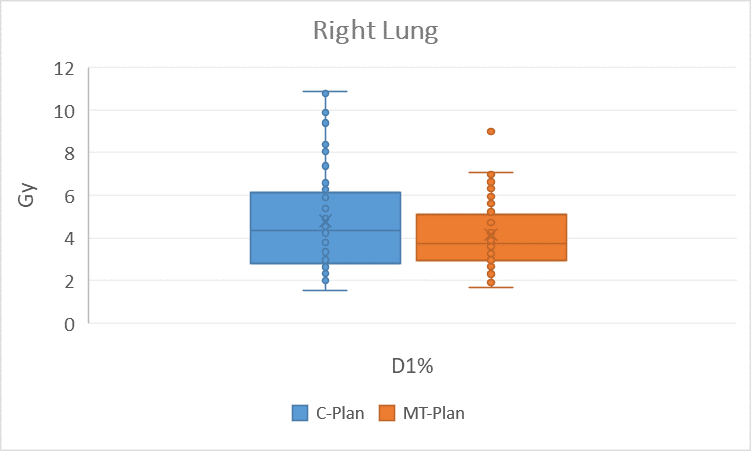


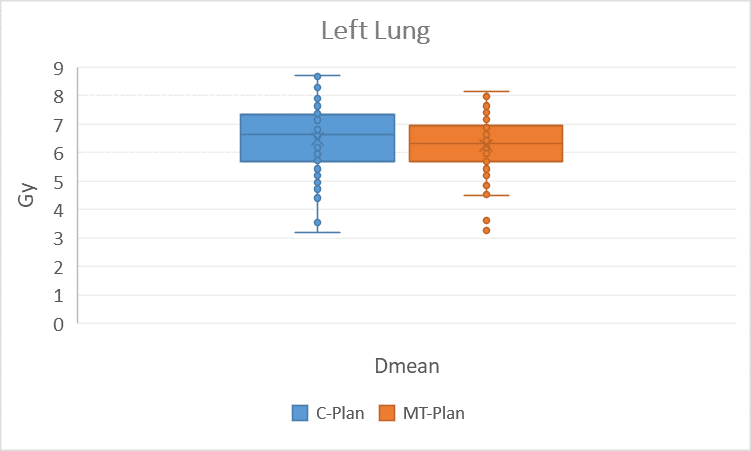


Figure 1s (b). Box plots dose comparison between the original clinical plans (C-Plans, depicted in blue) and plans used for model training (MT-Plans, depicted in orange) for OARs. The dots represent the clinical goal value for each plan, while the straight horizontal line, and the cross in the box represent the median and the mean values of the dose distributions (60 cases for each plan cohort), respectively.

Table 1s. Planning comparison of the original clinical plans before data curation (C-Plans) and model training curated plans (MT-Plans) evaluating PTVs and OARs mean dose (±SD). Dx represents the dose received by the x volume (in %), and Vy indicates the volume receiving the y relative (in %) or absolute (in Gy) isodose. The PTV2_Crop is the volume difference between PTV2 and PTV1. LAD refers the left-anterior-descending artery. The last row of the table presents the resulting p-value obtained from the Wilcoxon signed-rank test.

| Structure | Figure of Merit | Unit | Plans (n=60) Mean (SD) | | p- value |
| --- | --- | --- | --- | --- | --- |
|  |  |  | C-Plans | MT-Plans |  |
|  |  |  |  | |  |
| PTV_Boost | D98% | Gy | 56.8±0.7 | 56.9±0.7 | > 0.2 |
|  | D2% | Gy | 61.6±0.5 | 61.6±0.4 | > 0.2 |
|  | V95% | % | 97.4±1.3 | 97.6±1.2 | > 0.1 |
|  | V105% | % | 0.1±0.3 | 0.1±0.1 | > 0.2 |
| PTV_Breast | D98% | Gy | 46.4±0.7 | 46.2±0.7 | > 0.1 |
|  | V95% | % | 96.4±0.1 | 96.2±0.9 | > 0.1 |
| PTV_Crop | D1% | Gy | 58.7±0.9 | 58.9±1.0 | > 0.1 |
|  | V52.5Gy | % | 0.12±0.05 | 0.12±0.04 | > 0.1 |
| Left Lung | V5Gy | % | 32.6±6.6 | 31.0±5.4 | < 0.01 |
|  | V10Gy | % | 18.6±4.2 | 17.7±3.3 | < 0.01 |
|  | V20Gy | % | 9.5±2.7 | 9.2±2.4 | < 0.02 |
|  | V40Gy | % | 1.9±1.1 | 1.8±1.1 | > 0.2 |
|  | Dmean | Gy | 6.5±1.2 | 6.3±1.0 | < 0.01 |
| Right Lung | D1% | Gy | 4.8±2.4 | 4.2±1.6 | < 0.01 |
|  | Dmean | Gy | 1.2±0.6 | 1.0±0.4 | < 0.01 |
| Heart | D1% | Gy | 5.8±2.6 | 4.9±1.4 | < 0.01 |
|  | Dmean | Gy | 1.5±0.5 | 1.4±0.3 | < 0.01 |
| Right Breast | D1% | Gy | 7.4±3.5 | 6.7±2.6 | < 0.05 |
|  | Dmean | Gy | 1.7±0.6 | 1.6±0.6 | > 0.2 |
| LAD | Dmax | Gy | 9.7±4.4 | 7.7±2.9 | < 0.01 |


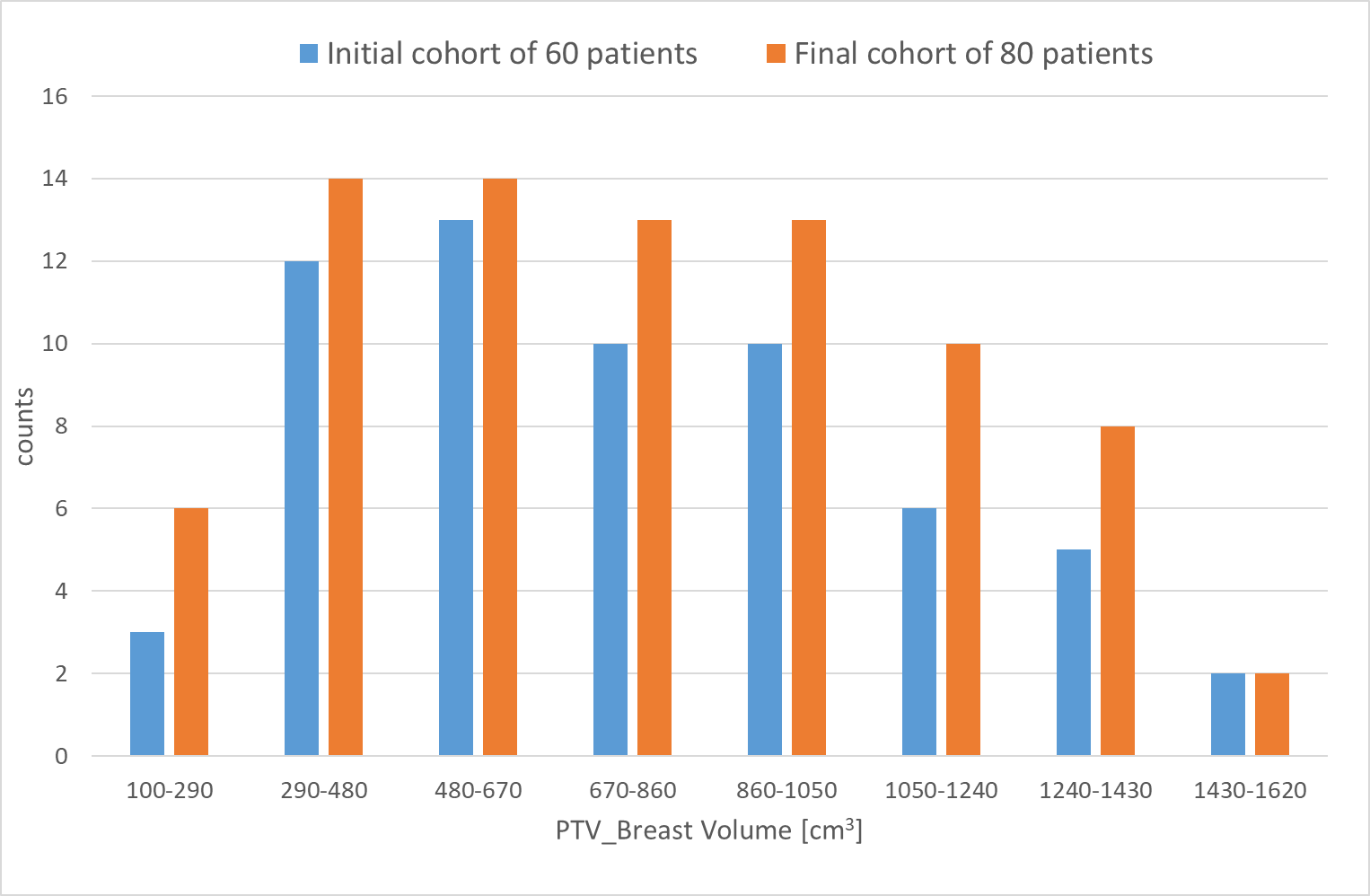


Figure 2s. The PTV_Breast volume distributions for the training data: the initial cohort of 60 patients (depicted in blue), and the final cohort used for model training, which comprises the initial 60 patients plus and additional 20 patients (represented in orange).

Table 2s. The qualitative evaluation of the different versions of the auto-planning model for the five test patients. Scores > 3 (range 1-5) indicate better plan quality for auto-plans.

|  | **Test Patient** | | | | | | | | | |
| --- | --- | --- | --- | --- | --- | --- | --- | --- | --- | --- |
| **Model Version** | **1** | | **2** | | **3** | | **4** | | **5** | |
|  | PTVs | OARs | PTVs | OARs | PTVs | OARs | PTVs | OARs | PTVs | OARs |
| v 1.0 | 2 | 5 | 1 | 5 | 1 | 4 | 2 | 3 | 3 | 3 |
| v 1.1 | 3 | 5 | 3 | 5 | 3 | 5 | 3 | 3 | 3 | 4 |
| v 1.2 | 3 | 5 | 4 | 5 | 3 | 5 | 3 | 3 | 4 | 4 |
| v 1.3 | 4 | 5 | 4 | 5 | 3 | 5 | 3 | 4 | 4 | 4 |


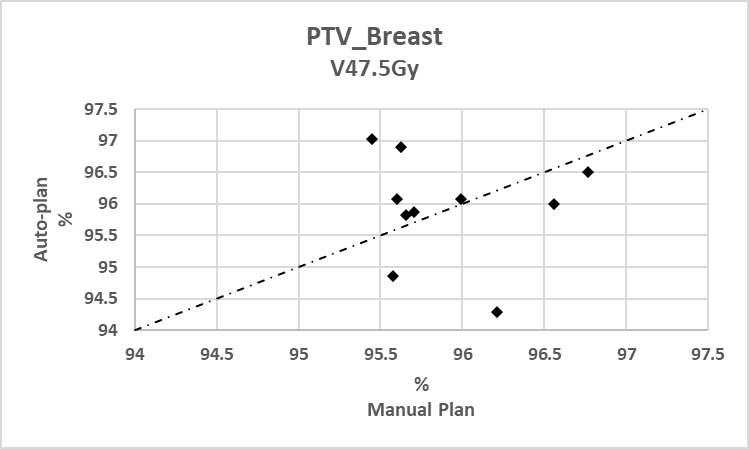

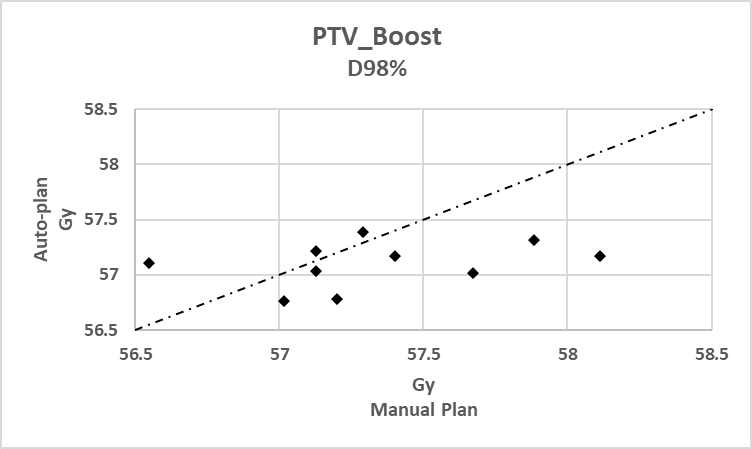


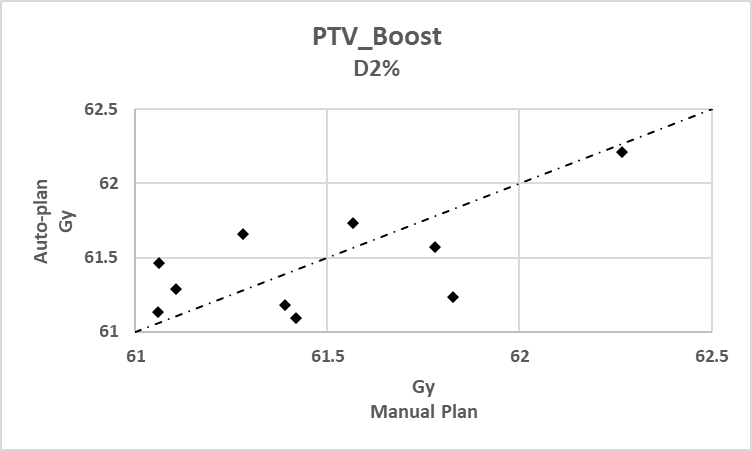


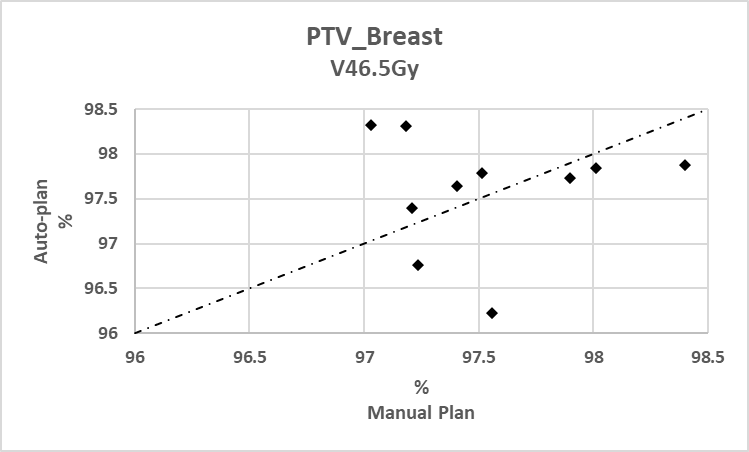


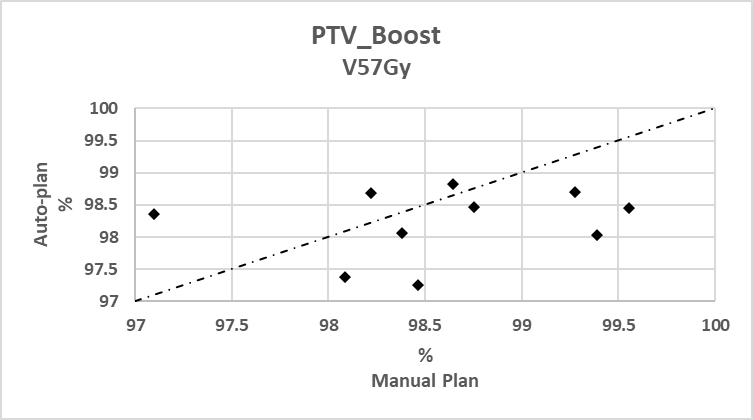


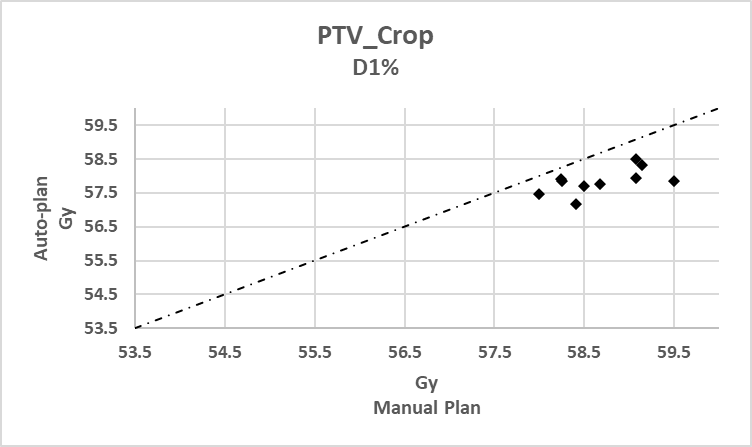


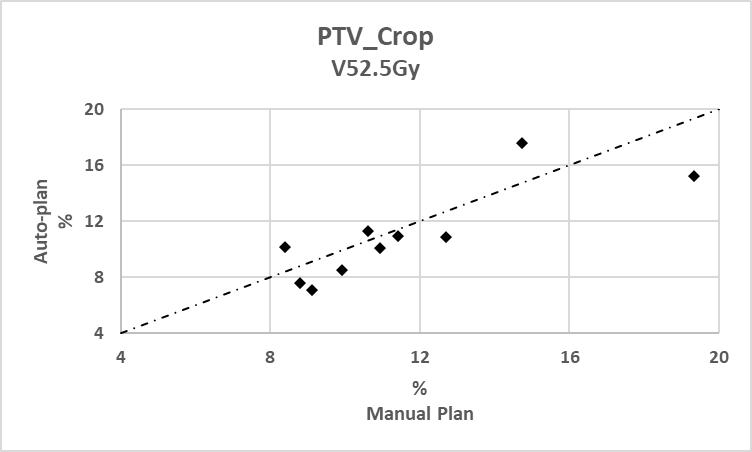

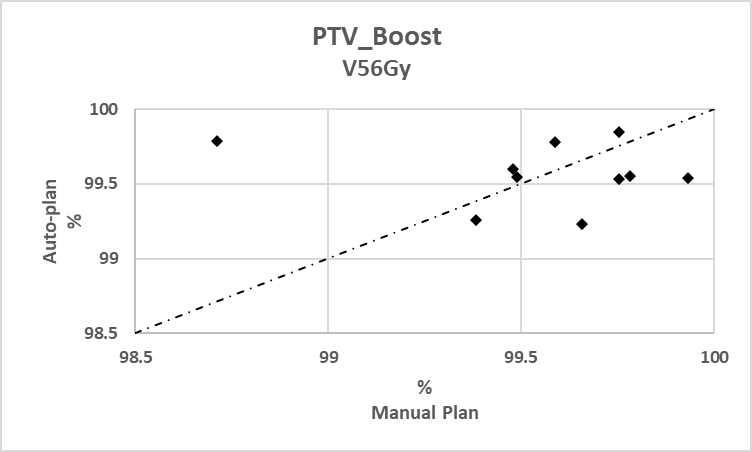


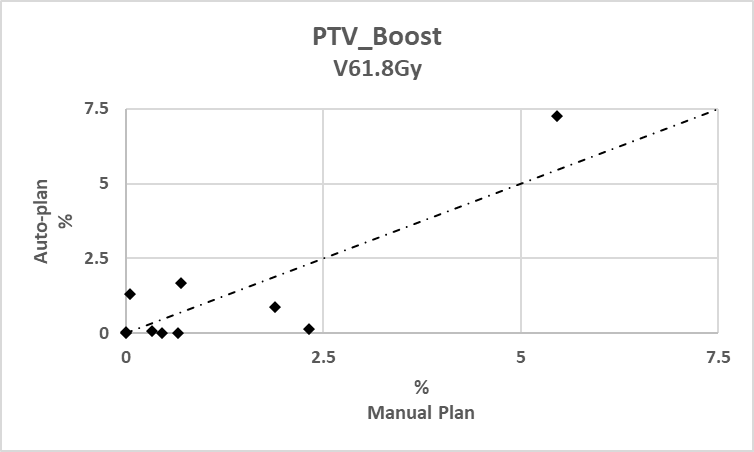


Figure 3s (a). Scatter plots of the 10 validation plans for targets. Automatic plans are plotted as a function of manual plans for the figures of merit evalauted. Dashed lines represent the equivalence between auto- and manual plans.


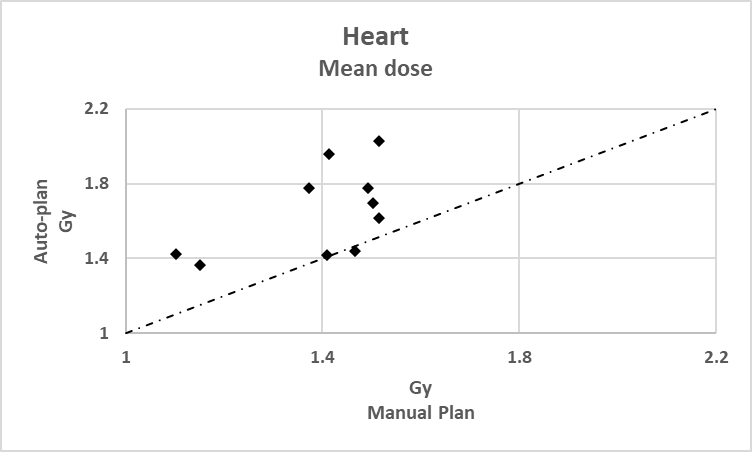

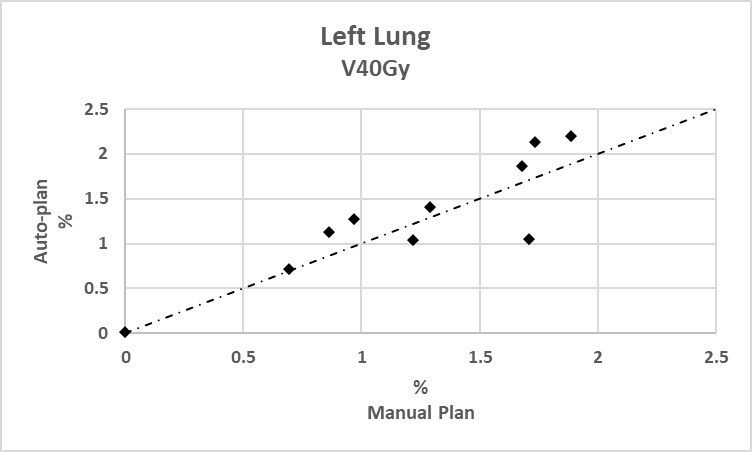

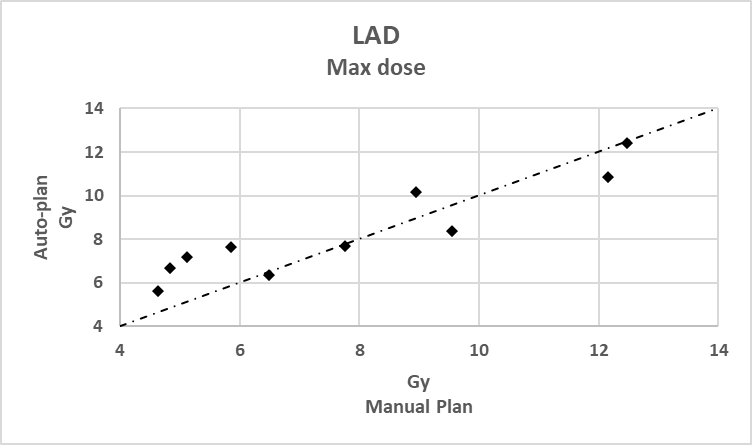

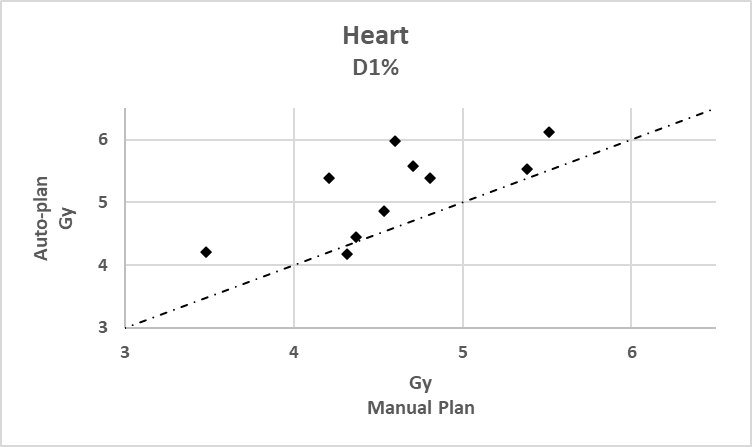

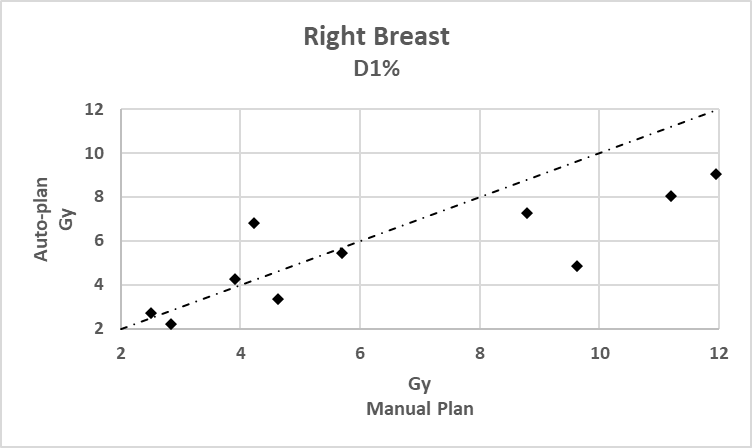

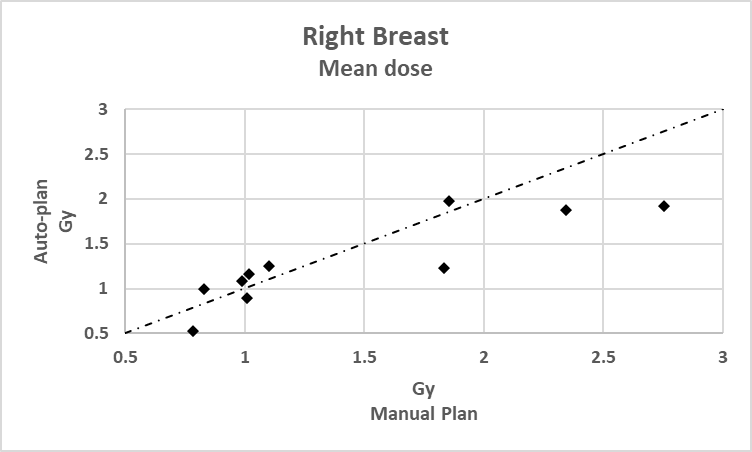

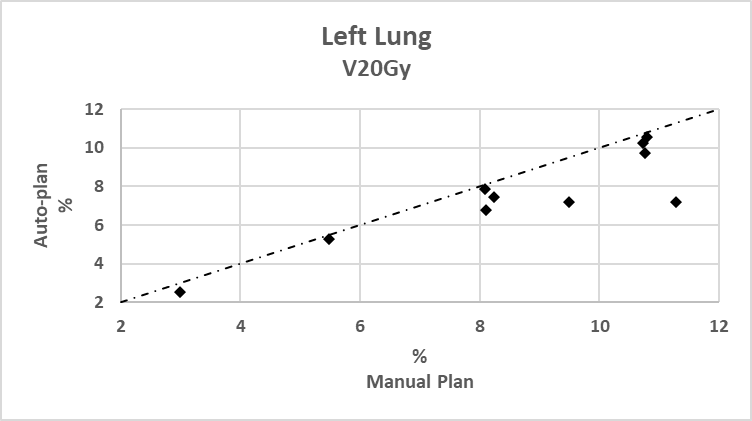

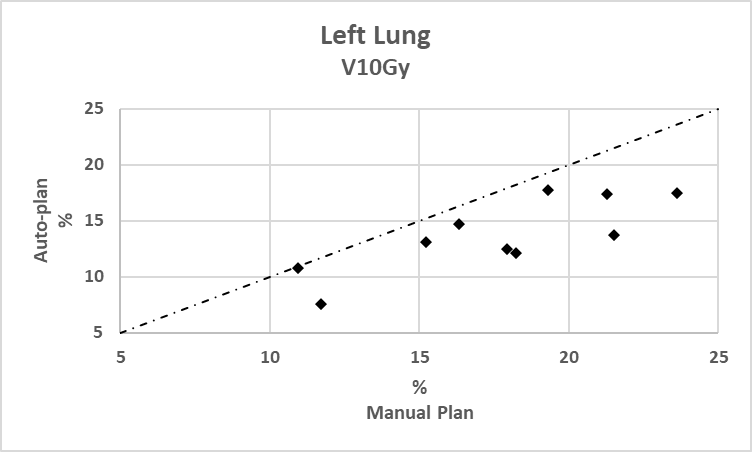

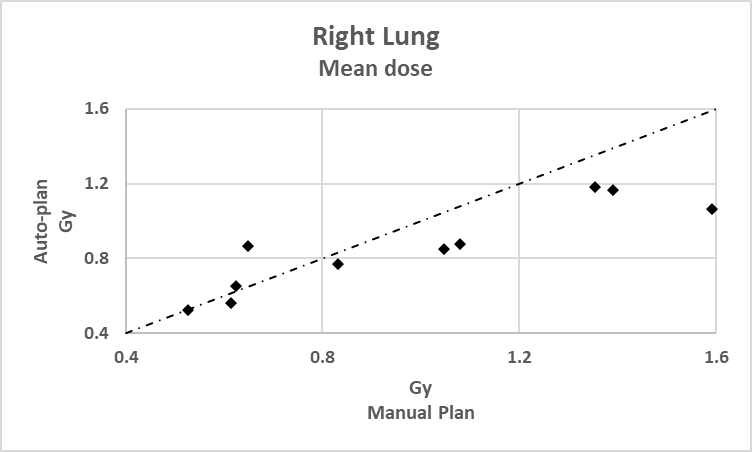

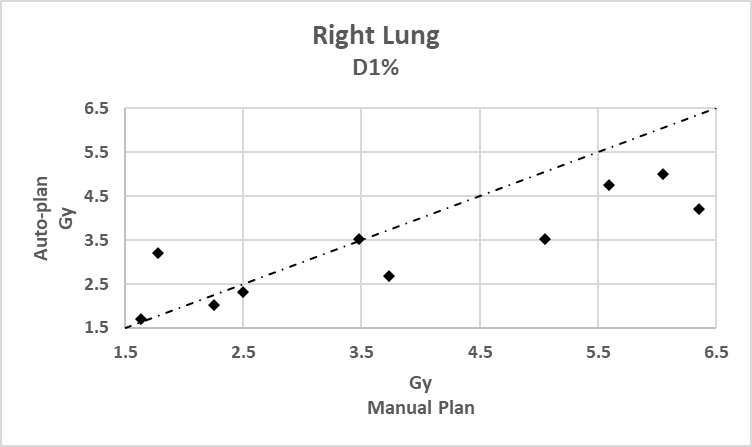

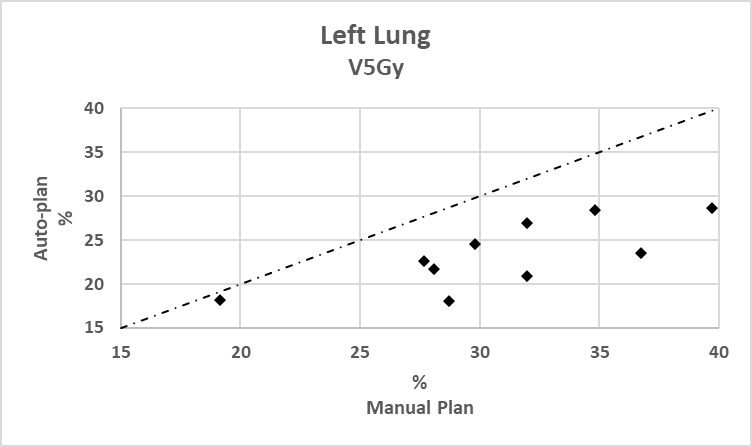


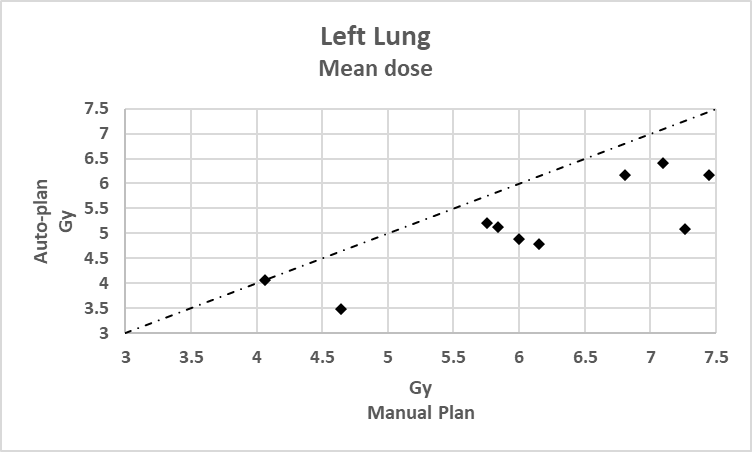


Figure 3s (b). Scatter plots of the 10 validation plans for OARs. Automatic plans are plotted as a function of manual plans for the figures of merit evaluated. Dashed line represents the equivalence between auto- and manual plans.

Table 3s. Evaluation of predicted and mimicked dose distribution against the clinical goal requirement specified in each model validation plan. Green dots represent results that meet the clinical goals, yellow dots indicate results within ±1% of the clinical goal requirement, and red dots signify results larger than ±1% of the clinical goal requirement.

Table 4s. Evaluation of clinical (i.e. mimicked) dose distribution against the clinical goal requirements specified in each of the 17 auto-plans clinically delivered. Green dots represent results that meet the clinical goals, yellow dots indicate results within ±1% of the clinical goal requirement, and red dots signify results larger than ±1% of the clinical goal requirement.
